# Dietary *Eucommia ulmoides* Extract Alleviates the Effect of Cold Stress on Chick Growth Performance, Antioxidant and Immune Ability

**DOI:** 10.3390/ani11113008

**Published:** 2021-10-20

**Authors:** Ting Hu, Yue Lei, Minxue Li, Qin Liu, Li Song, Degang Zhao

**Affiliations:** 1 Key Laboratory of Plant Resource Conservation and Germplasm Innovation in Mountainous Region (Ministry of Education), Guizhou University, Guiyang 550025, China; hut1020@163.com (T.H.); leiyue94@163.com (Y.L.); lmxzm95@163.com (M.L.); 2 Guizhou Key Lab of Agro-Bioengineering, Institute of Agro-Bioengineering, Guizhou University, Guiyang 550025, China; 3 College of Life Sciences, Guizhou University, Guiyang 550025, China; 4 Guizhou Institute of Subtropical Crops, Xingyi 562400, China; 5 College of Animal Science, Guizhou University, Guiyang 550025, China; lpslq95@gmail.com; 6 Guizhou Academy of Agricultural Science, Guiyang 550006, China

**Keywords:** *Eucommia ulmoides* extract, chick, cold stress, growth performance, antioxidant, immune

## Abstract

**Simple Summary:**

The cold stress that chicks suffer from has caused huge losses to the chicken industry. It is very important to find an effective way to alleviate cold stress in chicks. This study was designed to test the hypothesis that dietary *Eucommia ulmoides* extract alleviates the effect of cold stress on chick growth performance, antioxidants, and immune ability. Interestingly, *Eucommia ulmoides* extract supplementation improved the growth performance, antioxidant status, and immune response and reduced the organ damage of chicks caused by cold stress, which shows that *Eucommia ulmoides* extract has potent protective effects against cold stress. These findings will be very beneficial to break down the bottleneck issue of chick production caused by cold stress.

**Abstract:**

This study aimed to investigate the protective value of *Eucommia ulmoides* extract (EUE) on chicks under cold stress. A total of 21 compounds were identified in EUE using mass spectrometry (LC-MS). Ninety chicks were divided into a control group (CS) fed a basal diet and an experimental group supplemented with EUE, exposed to 10 ± 1 °C for 8 h per day. Results showed, compared with the CS group, the body weights (BW) (*p* < 0.01) and average daily gains ADG (*p* < 0.05) of the EUE group were increased throughout the study period. Chicks fed EUE had higher AFI (0–7 d, *p* < 0.001) and lower feed-to-gain ratios (F/G) (0–15 d, *p* < 0.001). EUE increased the activities of superoxide dismutase (SOD) (15 d, *p* < 0.05) and glutathione peroxidase (GSH-Px) (7 d, *p* < 0.05), whereas it decreased malondialdehyde (MDA) (15 d, *p* < 0.01). The contents of IgA (7 d, *p* < 0.05), IgG (7 d; 15 d, *p* < 0.01), and IgM (15 d, *p* < 0. 001) were higher in the EUE group. Dietary EUE could also reduce chick organ damage. Overall, EUE as a natural feed additive can improve the growth performance, antioxidant capacity, and immune level, and reduce the organ damage of cold-stressed chicks.

## 1. Introduction

Temperature is one of the major environmental factors for regulating animal health and survival [1]. It also affects production performance or reduces animal welfare. In critical environmental conditions, the vital physiological processes of homoeothermic animals are usually compromised [1]. Cold stress often occurs in animal management [2]. Low temperature has a negative influence on organisms, including leading to metabolic suppression [3], disrupting hormone release [4], and affecting the activity of protective enzymes [5], which can inhibit growth and increase mortality [2,6]. Low temperature also increases the immune-suppression level, free radical load, and leads to oxidative stress [7]. The animal immune system is often vulnerable to low temperatures and through the exacerbation of pathophysiological conditions [8]. Cold stress can also cause damage to the bursa of poultry, heart, intestinal tissue, and other slow development, thus affecting the growth of poultry and even causing death [9,10,11].

Low temperature is known as a major risk factor for diminishing the growth performance and survival of chicks [12]. Typical features of chicks include short villi, hypoplasia of organs, and an inadequate thermoregulatory system [13]. The hatching and brood temperature of chicks is higher than most other animals such as cattle and sheep. Chicks just out of the shell are highly susceptible to low temperatures, and once the ambient temperature is not sufficient for the chick to grow, this often leads to a high mortality rate [14]. Low temperature also reduces chicken antioxidant capacity [15,16], immune function [17], and damages tissues and organs in chicken [18]. In addition, low temperature can reduce appetite, feed intake, and feed utilization of chicks, which can affect growth performance and product quality [14]. Low temperatures tend to limit poultry production, especially causing serious economic losses to chick production, which is a bottleneck that needs to be solved [15].

Traditional measures to reduce the impact of low-temperature stress on chicks include increasing padding, raising the environmental temperature, adding vitamins and antibiotics, etc., [19,20,21], which are costly, energy consuming, and environmentally unfriendly [20]. Using medicinal plant additives has been one of the common trends to improve the low-temperature stress on chicks in recent years [22,23,24]. Feeding Chinese herbal medicines (CHM) can promote animal growth, enhance antioxidant levels, and enhance immune ability [23,25], in order to reduce the impact of environmental stress on the animal.

*Eucommia ulmoides* is a precious traditional CHM. Its bark, leaves, and flowers have various active ingredients, which can be used as medicine [26]. The medicinal value of *Eucommia ulmoides* has attracted more and more attention for its chemical composition [27]. *Eucommia ulmoides* is rich in chemical components, such as iridoids, flavonoids, phenylpropanoids, lignans, and so on [19,28,29]. *Eucommia ulmoides* has a variety of biological activities, including growth promotion [30], metabolic regulation [28], antioxidant abilities [31], immune enhancement [32], anti-inflammatory abilities [28], and other effects. Chlorogenic acid, flavonoid, and rutin in *Eucommia ulmoides* have an obvious effect of promoting growth [33,34,35]. Chlorogenic acid, flavonoid, and quercetin have an antioxidant effect [36,37,38,39]. Furthermore, flavonoids can improve the immune ability of the body [40]. Studies have reported that *Eucommia ulmoides* and its extracts can be used as feed additives, which have the functions of improving growth performance, antioxidant capacity, and immune response [41,42]. However, to the best of our knowledge, very few studies have been conducted using *Eucommia ulmoides* or its extracts for animal cold stress protection. In this study, *Eucommia ulmoides* leaves (EUL) were used as raw materials to extract the active components and fed to chicks under cold stress, and their growth performance, organ development, serum antioxidant capacity, and immunoglobulin content were determined. The aim of the present study was to explore the effects of EUE on chicks in low-temperature environments, so as to provide a scientific basis for the application of EUE as a feed additive to protect cold-stressed chicks.

## 2. Materials and Methods

All procedures of animal experiments were performed according to the protocol approved by the Guizhou University Subcommittee of Experimental Animal Ethics (EAE-GZU-2020-P020, Guizhou, China). The chicks used in this study have been treated humanely. Great efforts have been made to minimize pain.

### 2.1. Preparation of EUE

EUL was picked from the South Campus of Guizhou University. The *Eucommia ulmoides* were dried at 80 °C and crushed by an ultrafine grinder. After being passed through an −80 mesh sieve, the powders were stored at −20 °C. The powders were supplemented with 10 volumes of distilled water and soaked at room temperature for 2 h accompanied by stirring several times. Then, the mixtures were extracted in a water bath at 80–90 °C for 1 h and centrifuged at 10,000 rpm for 5 min at 25 °C. The supernatants were dried in an oven at 60 °C to obtain EUE. Fresh EUL were dried and crushed into EUL powder.

### 2.2. Composition Determination of EUL and EUE by LC-MS

Sample preparations. First, 0.2 g EUE and EUL powders were respectively placed in 10 mL centrifugal tubes. The samples were ultrasonically extracted with 50 mL of 8% methanol for 40 min at 45 °C and left to rest for 5 min. The supernatants were removed, placed in new centrifuge tubes and centrifuged at 13,000 rpm for 10 min, and filtered with a 0.22 µm microporous membrane to obtain the extractions, which were stored in a refrigerator at 4 °C.

Chromatographic conditions. Extractions were injected into a C18 column (100 mm × 2.1 mm, 1.8 μm) at a 0.3 mL/min flow rate. The column temperature was set at 35 °C and the sample volume was 5 μL. Gradient elution was performed with mobile phases A (0.1% formic acid aqueous solution) and B (acetonitrile) by HPLC (LC-30A, Shimadzu, Kyoto, Japan). The elution procedure was shown in Table 1.

Mass Spectrometry Conditions. Electrospray ionization was used in the positive ion mode with the following details: Temperature, 600 °C; spray voltage, 5500 V; declustering potential (DP), 100 V; collision energy (CE), 35 eV; collision energy spread (CES), 15 eV; nebulizing gas, nitrogen; curtain gas, 35 psi; auxiliary gas1, 60  psi; auxiliary gas 2, 50 psi; mass scan, 50 to 1000 m/z. Electrospray ionization was used in the negative ion mode: Spray voltage, −4500 V; collision energy (CE), −35 eV. The rest of the experimental conditions remained in positive ion mode.

### 2.3. Animals and Experimental Design

The animal experiment design was referenced from previous literature [43]. A total of 90 2-week-old healthy chicks (iron-footed hemp variety) with similar BWs were randomly divided into two equal groups (45 chicks each group), one of which was randomly selected as the control group and the other as the treatment group. Then, chicks were randomly assigned to 3 stages of the experiment, which were 0 d, 7 d, and 15 d, respectively. Each pen had 5 chicks and 3 replicate pens. Chicks in each pen were reared in the same breeding house equipped with a feeder, an automatic drinker, and straw pellets as bedding to ensure the same feeding environment. The control group was fed with a basal diet (CS) using the Broiler chicken compound diet 510 (Guiyang Special Drive Hope Agricultural Science and Technology Co., LTD, Guiyang, China). In addition, the treatment group was fed a basal diet supplemented with 0.8% EUE according to the relevant literature [37,44]. The basal diet ingredients are presented in Table 2. In order to investigate the repeated response of the chicks to the low-temperature environment and avoid long-term low-temperature stress leading to the death of the chicks, the environmental temperature control design was carried out according to the relevant literature [45]. Chicks of the above two groups were exposed to 10 ± 1 °C from 22:00 to 6:00 to simulate cold stress, and returned to a normal temperature at 25 ± 1 °C from 6:00 to 22:00; this lasted for 15 d. The relative humidity was 65 ± 5% and the light:dark cycle was 10 h:14 h per day. Water and feed were provided ad libitum to the chicks.

### 2.4. Growth Performance

The BW and feed intake (FI) of each replicate were recorded at 0 d, 7 d, and 15 d. The ADG, average feed intake (AFI), and F/G were calculated for 0–7 d, 8–15 d, and 0–15 d, respectively. The mortality rate was recorded daily and was used to calculate the mortality-corrected FCR.

### 2.5. Antioxidant Index and Malondialdehyde Content

At 0 d, 7 d, and 15 d, 5 chicks in each repeated group were slaughtered and sampled (15 chicks in each group, with a total of 90 chicks). Blood was collected from the jugular vein and placed in the procoagulant tube. After standing for 1–2 h at 4 °C, the samples were centrifuged for 10 min at 4 °C (3000 rpm) to separate the serum. Five chicks’ serum from each replicate group was mixed (in equal quantity) (*n* = 15) [46,47]. The levels of total antioxidant capability (T-AOC), superoxide dismutase (SOD), glutathione-peroxidase (GSH-Px), and malondialdehyde (MDA) in serum were individually determined using the protein or MDA enzyme-linked immunosorbent assay (ELISA)kits (Nanjing Jiancheng Bioengineering Institute, Nanjing, China). The operation was carried out according to the manufacturer’s instructions and repeated three times.

### 2.6. Immune Index

The levels of IgA, IgG, and IgM contents in chick serum were measured at 0 d, 7 d, and 15 d. The collection procedure of the serum sample was the same as above (*n* = 15). Total IgA, IgG, and IgM in serum were respectively determined by immunoglobulin ELISA kits (Shanghai Jining Biological Technology Co., Ltd., Shanghai, China). The operation was carried out according to the manufacturer’s instructions and repeated three times.

### 2.7. Determination of Organ Index

One chick from each replicate representing the mean weight of the pen was selected, slaughtered, and eviscerated (*n* = 3). The liver, heart, spleen, thymus, bursa of Fabricius (BF), duodenum, and musculature were separated and sucked with filter paper. After the blood had dried, their weights were valued, and the organ indexes were then calculated as follows [48]: Organ index = organ weight (g)/body weight (g) × 100%.

### 2.8. Histopathological Examination

When slaughtered at 15 d, heart, liver, and duodenum samples were collected from 1 chick in each replicate for histological analysis (*n* = 3). The tissues were fixed in tissue fixative (Wuhan Saiweier Biological Technology Co., Ltd., Wuhan, China) for 24 h and later dehydrated by consecutive washes with ethyl alcohol (70–100%). Subsequently, xylol was used to diaphanize the samples, then embedded in paraffin wax, and serial paraffin sections (4 μm) were obtained. The sections were cut by a microtome and fixed on slides. Sections were stained with hematoxylin and eosin (H&E) (Beyotime, Shanghai, China) and then observed under a microscope.

### 2.9. Statistical Analysis

Data analysis, single-factor ANOVA, and Duncan’s multiple comparisons were performed using Microsoft Excel Tools and SAS 9.4 software (SAS Institute Inc., Cary, NC, USA) for comparing the differences in growth performance, antioxidant, and immune ability between the EUE group and control group. Results were presented as mean values ± standard deviation (SD). Probability values of *p <* 0.05 were considered statistically significant.

## 3. Results

### 3.1. The Composition of EUE and EUL

A total of 21 compounds were putatively identified in the EUE and EUL using LC-MS analysis, including six iridoids, five flavonoids, four Phenylpropanoids, two lignans, and four others (Table 3). Results showed that the contents of active ingredients in the extract were all higher than that of leaves. Iridoids were abundant in the extract, among which the highest content of genipin was 56.2 mg/g, followed by asperulosidic acid with 50.5 mg/g, was well as aucubin with 17.1 mg/g, eucommiol with 1.71 mg/g, catalpol with 1.53 mg/g, and eucommiside with 0.560 mg/g. The content of the flavonoids kaempferol, isoquercitrin, kaempferol-3-O-rutinoside, quercetin, and rutin in EUE was 21.8 mg/g, 1.68 mg/g, 21.8 mg/g, 1.68 mg/g, 1.36 mg/g, 0.960 mg/g, and 0.110 mg/g respectively, and their contents were about 10 times that of EUL. Phenylpropanoids including chlorogenic acid, methyl chlorogenate, neochlorogenic acid, and cryptochlorogenic acid were all found in both EUE and EUL. Among them, the content of chlorogenic acid was the highest, at 36.8 mg/g in EUE but only 2.84 mg/g in EUL. The lignin substances of terpineol and 8-Hydroxypinoresinol were also detected in two samples. The EUE possessed a high content of 36.7 mg/g of total isomaltose compared to EUL (3.60 mg/g). In addition, 5-Hydroxymethylfurfural, caffeic acid, and medioresil were also detected in EUE and EUL. Results indicated that EUE contained the same active ingredients as EUL. Moreover, the contents of active ingredients of EUE were all higher than EUL.

### 3.2. Growth Performance under Cold Stress

According to Table 4, the initial BW of chicks did not differ (*p* > 0.05) between dietary treatments. After being fed EUE, the BW of chicks at 7 d and 15 d was significantly higher than that of the control group by 65.0 g (*p* = 0.001) and 78.0 g (*p* < 0.001), respectively. The chicks fed EUE had a greater average daily weight gain (ADG) than control chicks from days 0 to 7 (*p* < 0.001) and 0 to 15 (*p* < 0.001). It has been demonstrated that feeding EUE can significantly increase the BW and ADG of chicks under cold stress.

The AFI and F/G of chicks under cold stress are shown in Table 4. The AFI in the EUE group was higher than that of the CS control group between days 0 and 7 (*p* < 0.001). However, the AFI of the EUE group was lower than that of the control group between days 8 and 15 (*p* = 0.001). It was observed that the EUE extractive feeding was beneficial for the increase in feed intake at the early stage (0–7 d), and may cause a decrease in intake over 8–15 d. It appeared that chicks in the group fed the extract gained a lower feed-to-gain ratio (F/G) than the controls under cold stress (Table 5). The F/G of chicks fed the extract were significantly lower by 32.8%, 9.70%, and 21.7% than the control chicks at 0–7 d (*p* < 0.001), 8–15 d (*p* < 0.01), and 0–15 d (*p* < 0.001). Results showed EUE could reduce the chicks’ F/G and improve the feed efficiency under cold stress.

### 3.3. Antioxidant Parameters under Cold Stress

The level of T-AOC, SOD, GSH-Px, and MDA of chick supplied different feeds were detected on 0 d, 7 d and 15 d (Table 6). They were no significant differences among the groups before cold stress exposure. However, these indicators changed significantly in feeding at 7 d or 15 d. The T-AOC of the EUE group increased by 13.4% (*p* < 0.05) and 24.4% (*p* < 0.05) at 0–7 d and 0–15 d, respectively. In the CS group, T-AOC increased by 6.86% at 0–7 d (*p* < 0.05) and 10.1% at 0–15 d (*p* < 0.05), showing a higher change than the EUE group. Similarly, The SOD of the EUE group (increased by 11.4% and 29.5%, *p* < 0.05) also presented a lower change than the CS group (increased by 7.91% and 21.1%, *p* < 0.05) at 0–7 d and 0–15 d. In addition, the SOD of the EUE group was significantly higher than the CS group at 15 d (*p* < 0.05). The GSH-Px of the EUE group (reduced by 7.08% and 17.5%, *p* < 0.05) had a lower rate of decline than the CS group (reduced by 16.6% and 19.5%, *p* < 0.05) at 0–7 d and 0–15 d. Meanwhile, The GSH-Px of the EUE group was 12.3% higher than the CS group at 7 d (*p* < 0.01). The MDA of the EUE group was significantly lower by 22.5% than the CS group at 15 d (*p* < 0.01).

### 3.4. Immune System Traits under Cold Stress

The IgA, IgG, and IgM in serum of 0 d, 7 d, and 15 d chicks were detected and presented in Table 7. The IgA of the EUE group was clearly higher by 6 μg/mL than the control group at 7 d (*p* < 0.05). The IgG of the EUE group significantly increased by 93.0 μg/mL (*p* < 0.01) and 280 μg/mL (*p* < 0.001) when compared to the CS group at 7 d and 15 d. The IgM of the EUE group was higher by 44.0 μg/mL (*p* < 0.001) than the CS group at 15 d.

### 3.5. Organ Development and Histopathology under Cold Stress

According to experimental results, the EUE group tended to have higher organ indexes, including heart, liver, spleen, BF, stomach, duodenum, and pancreatic, than the control group at 7 d and 15 d, except at 7 d for the spleen index. Unfortunately, these differences were not significant (Table 8).

**Figure 1:**
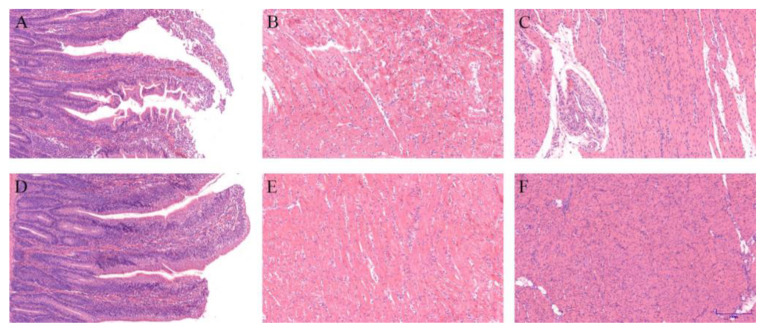
H&E staining tissue sections of duodenum, heart, and stomach. (**A**–**C**) CS group (**A**): Duodenum; (**B**): Heart; (**C**): stomach. (**D**–**F**) EUE group (**D**): Duodenum; (**E**): Heart; (**F**): Stomach.

Results indicated that cold stress could damage the duodenum, heart, and stomach of chicks at 15 d, clearly elucidating the disrupted duodenal villi structure and inflammation in the duodenum (Figure 1A,D). It was also observed that myocardial fibers were disordered and broken in the heart (Figure 1B,E) and stomach muscles, and fiber tissues were broken and erosion spots appeared (Figure 1C,F). However, the duodenum, heart, and stomach tissues of chicks in the EUE group were less damaged than the CS group.

## 4. Discussion

*Eucommia ulmoides* is one of the traditional CHMs, which contains a variety of active ingredients in its root, bark, leaf, and flower, with multiple economic and pharmacological values [49,50,51]. The identification of *Eucommia ulmoides* and their extracts have been a hot spot in the research and utilization of *Eucommia ulmoides*. Our study showed that the water extract of EUL contained lignans, iridoids, phenylpropanoids, flavonoids, and so on, which is consistent with that extracted by organic solvent [52,53]. However, the water extract has the advantages of low cost, simple operation, and less pollution. It has been reported that iridoids, flavonoids, phenylpropanoids, and other substances in *Eucommia ulmoides* have antioxidant, anti-inflammatory, and immunity effects [28,54,55]. In addition, chlorogenic acid [36,37], quercetin [38], and rutin [56] increase immune and antioxidant capacity and promote animal growth and development. It was shown that EUE contained abundant chlorogenic acid, galangal, woodruff nucleoside acid, peach leaf coral glycosides, rutin, and quercetin, which may improve the immune function and growth performance of chicks, indicating EUE can be a potential feed additive.

Cold stress can reduce the growth performance of animals, such as BW and feed efficiency [57,58,59]. In the present study, the chicks supplemented with EUE showed better growth performance of higher BW (*p* < 0.01) and ADG (*p* < 0.05) and lower F/G (*p* < 0.001) throughout the study period, and higher AFI at 0–7 d (*p* < 0.001). Our study showed EUE had a better protective effect on cold-stressed poultry. Studies have shown that feeding *Eucommia ulmoides* can improve animal growth performance [60]. Previous investigations found that after feeding *Eucommia ulmoides* (rich in chlorogenic acid), the weight of grass carp increased by 5.22%, indicating that *Eucommia ulmoides* can promote the growth of grass carp [34]. It was reported earlier that adding 1 g/kg rutin can improve the growth performance of broilers [61]. Additionally, the addition of EUE containing flavone improved the growth performance of piglets stimulated by dipterous [35]. The reason EUE can protect chicks under cold stress may be that EUE contains high levels of chlorogenic acid, rutin, flavonoids, etc., which promotes the BW, ADG, and AFI of chicks, decreases the F/G, and alleviates the impact of cold stress.

The body’s antioxidant level reflects the ability to resist stress [62]. T-AOC, SOD, and GSH-Px in serum are all essential components of the antioxidant defense system, and they have very significant impacts on homeostasis between oxidation and antioxidants [63]; MDA is one of the end-products of lipid peroxidation, and its level reflects the degree of oxidative injury to the organism [64]. As reported, cold stress could cause an increase in T-AOC, SOD, and GSH-Px levels [65] and the reduction of MDA levels [66]. Previous studies reported that EUE improved the antioxidant capacity of weaned piglets [31,67]. Additionally, the dietary chlorogenic acid-enriched extract (CGAE) from *Eucommia ulmoides* supplementation improved oxidative status in pigs [60]. Bai et al. (2019) demonstrated that the addition of *Eucommia ulmoides* to diet could significantly increase GSH-Px activity and decrease MDA content, thus improving the antioxidant capacity of hemp ducks [41]. Chlorogenic acid and flavonoids are both natural antioxidants [68,69]. The chlorogenic acid-enriched extract (CGAE) from *Eucommia ulmoides* leaves significantly elevated T-AOC and SOD activity in the liver and serum of lambs under stress, reduced MDA content, improved antioxidant status, and alleviated oxidative damage of lambs [42]. The literature also shows that EUE (flavonoids) alleviates the oxidative stress induced by diquat in piglets by reducing the growth performance impairment, pro-inflammatory cytokines secretion, and intestinal barrier dysfunction [35]. In this study, the results showed that chicks fed with EUE could significantly increase the levels of SOD at 15 d (*p* < 0.05) and GSH-Px at 7 d (*p* < 0.01), and significantly reduce the level of MDA at 15 d (*p* < 0.01). Thus, EUE can reduce the damage of cold stress to the chicks by improving the antioxidant ability, which may be related to chlorogenic acid and flavonoids in the antioxidant substances in EUE.

The variations of ambient temperature affect the immune response of chicks [70,71]. The immune system is often subject to low temperatures, which reduces the activity of immunoglobulins such as IgA, IgG, and IgM, resulting in immunosuppression in animals [72]. A previous study has shown that dietary EUL extracts significantly increase the blood IgG and IgM contents of weaned piglets [73] and broilers [74]. Additionally, Flavonoids extracted from Chinese herbs could improve immune function and alleviate immune stress in broilers challenged with lipopolysaccharide [75]. An animal’s systemic immune status may be reflected by the concentration of IgA, IgG, and serum IgM [76]. In the present study, the level of IgA (7 d, *p* < 0.05), IgG (7 d, *p* < 0.01; 15 d, *p* < 0.001), and IgM (15 d, *p* < 0. 001) in the EUE group were significantly higher than that in the CS group at the same stage under low temperatures. Our study suggests that EUE may enhance chicks’ immune function by increasing the serum immunoglobulin contents (IgA, IgG, and IgM), thus having a protective effect against cold stress on chicks.

The organ index often reflects the relative growth and functional state of organs [77]. The liver, spleen, and BF are important immune organs, and their organ indexes could reflect the immune function [78,79,80,81,82]. EUL extract could increase the liver index and contribute to the alleviation of the weaning stress response in piglets [64,83]. In addition, EUL extract can increase the index of spleen and BF and improve the immune function of hemp ducks [41]. The work presented here showed that EUE had no significant effects on the indexes of heart, liver, spleen, BF, stomach, duodenum, and pancreas of chicks under cold-stress conditions. Interestingly, we observed that, compared with the control group, the EUE group had a certain degree of improvement in all organ indexes except the spleen at 7 d. To a certain extent, feeding EUE could reduce the effects of cold stress on the organ development of chicks. Unfortunately, the differences were not significant. The results may be related to the fact that only 0.8% EUE was added in this experiment.

The heart, duodenum, and stomach are essential organs for metabolism and nutrient absorption, and their health degree reflects the adaptability of an organism to environmental stress [84,85]. It has been reported that stresses such as cold exposure can induce heart, duodenum, and stomach injury [9,86]. In the present study, cold stress caused damage to the tissue of the heart, duodenum, and stomach by H&E staining, while the damage in the EUE group was milder than the CS group. EUL extract can reduce weaned pigs’ intestinal damage caused by stress [87]. Consistent with the aforementioned reports, we identified that EUE could reduce the organ damage of chicks under cold stress, which may be related to the EUE elevating metabolism and nutrient absorption and enhancing organ health.

## 5. Conclusions

In summary, this study showed that EUE had obvious protective effects against chicks’ cold stress. The dietary EUE supplementation improved the growth performance, antioxidant, and immune ability of chicks, and reduced organ damage caused by cold stress, to realize the protective effect of a water extract from *Eucommia ulmoides* on cold-stressed chicks. These findings can provide a new control strategy for the bottleneck issue of chick production caused by cold stress, although further studies are needed to further explain the protection mechanism.

## Figures and Tables

**Table 1:** The elution program.

Time (min)	A (0.1% Formic Acid Aqueous Solution)	B (Acetonitrile)
0.010	95.0	5.00
15.0	75.0	30.0
30.0	5.00	95.0
32.0	5.00	95.0
32.1	95.0	5.00
35.0	stop	

**Table 2:** Ingredient composition and calculated nutrient content of the basal diets.

Ingredient, %		Nutrients ^2^, %	
corn	58.5	Metabolizable energy, kcal/kg	3130
Wheat bran	3.20	Crude protein	22.1
Soybean meal	27.5	Crude fiber	3.56
fish meal	3.10	Crude ash	6.80
Soyabean oil	4.22	Total phosphorus	0.690
Dicalcium phosphate	2.14	Available phosphorus	0.530
Sodium chloride	0.420	Methionine	0.430
DL-methionine	0.350	Calcium	1.06
L-Lysine·HCl	0.270	Met	0.430
Vitamin/mineral premix ^1^	0.300		
Total	100		

^1^ Provided the following nutrients per kg of diet: Vitamin A, 6000 IU; vitamin D3, 800 IU; vitamin E, 20 IU; vitamin K3, 2 mg; vitamin B2, 2 mg; Cu, 21 mg; Fe, 100 mg; Zn, 60 mg; Mn, 90 mg; I, 1.0 mg; Co, 0.3 mg and Se, 0.3 mg. ^2^ All data are the results of chemical analysis con-ducted in triplicate.

**Table 3:** Components and contents of EUE and EUL.

Components	Compounds	EUE	EUL
		Contents (mg/g)	
Iridoids	Genipin	56.2	5.81
	Asperulosidic acid	50.5	5.42
	Aucubin	17.1	1.14
	Catalpol	1.53	0.090
	Eucommiol	1.71	0.090
	Eucommiside	0.560	0.040
Flavonoids	Kaempferol	21.8	2.12
	Isoquercitrin	1.68	0.120
	Kaempferol-3-O-rutinoside	1.36	0.080
	Quercetin	0.960	0.060
	Rutin	0.110	0.007
Phenylpropanoids	Chlorogenic acid	37.0	2.84
	Methyl chlorogenate	0.510	0.030
	Neochlorogenic acid	0.480	0.030
	Cryptochlorogenic acid	0.240	0.020
Lignans	Terpineol	2.60	0.130
	8-Hydroxypinoresinol	0.900	0.050
Other compounds	Isomaltose	36.7	3.56
	5-Hydroxymethylfurfural	2.47	0.130
	Caffeic acid	0.280	0.020
	Medioresil	1.44	0.120

**Table 4:** The weight change of chicks under cold stress.

Group	Body Weight (g)			Average Daily Gain (g)		
	0 d	7 d	15 d	0–7 d	8–15 d	0–15 d
CS	200 ± 8.16 ^a^	310 ± 8.16 ^b^	493 ± 4.71 ^c^	13.8 ± 0.000	22.9 ± 0.590	18.3 ± 0.290
EUE	198 ± 2.36 ^a^	375 ± 4.08 ^b^*	572 ± 8.50 ^c^*	22.1 ± 0.290 *	24.6 ± 0.590	23.3 ± 1.40 *
*p*-value	0.7952	0.0005	0.0003	<0.0001	0.0474	0.0001

Symbol * indicates a significant difference at *p* < 0.05 between the *Eucommia ulmoides* group and the control group at the same time. ^a,b,c^ Values with different letters within the same row are significantly different (*p* < 0.05). Values are the mean ± SEM. Results are means of 15 samples obtained from the test chicks of each control and experimental group.

**Table 5:** The AFI and F/G of chicks under cold stress.

Group	Average Feed Intake (g)			Feed to Gain Ratio		
	0–7 d	8–15 d	0–15 d	0–7 d	8–15 d	0–15 d
CS	43.5 ± 0.160	61.4 ± 0.650	52.4 ± 0.850	3.17 ± 0.020	2.68 ± 0.070	2.86 ± 0.050
EUE	47.2 ± 0.010 *	57.2 ± 0.050 *	52.2 ± 1.13	2.13 ± 0.030 *	2.42 ± 0.060 *	2.24 ± 0.040 *
*p*-value	0.0001	0.0010	0.7908	<0.0001	0.0050	0.0001

Symbol * indicates a significant difference at *p <* 0.05 between the *Eucommia ulmoides* group and the control group at the same time. Values are the mean ± SEM. Results are means of 15 samples obtained from the test chicks of each control and experimental group. AFI: Average Feed Intake; F/G: Feed-to-Gain Ratio.

**Table 6:** The oxidative and antioxidative indicators in chick serum under cold stress.

Index	Group	0 d	7 d	15 d
T-AOC (U/mL)	CS	9.04 ± 0.110 ^a^	9.66 ± 0.150 ^a^	9.95 ± 0.910 ^a^
	EUE	9.08 ± 0.150 ^a^	10.3 ± 0.310 ^b^	11.3 ± 0.210 ^c^
*p*-value	/	0.7638	0.0552	0.1092
SOD (U/mL)	CS	83.4 ± 0.240 ^a^	90.0 ± 3.12 ^b^	101 ± 1.72 ^c^
	EUE	82.6 ± 0.950 ^a^	92.0 ± 2.89 ^b^	107 ± 1.95 ^c^*
*p*-value	/	0.2192	0.4770	0.0126
GSH-Px (U)	CS	421 ± 7.22 ^a^	351 ± 6.52 ^b^	339 ± 11.2 ^c^
	EUE	424 ± 7.77 ^a^	394 ± 9.41 ^b^*	350 ± 5.77 ^c^
*p*-value	/	0.7330	0.0029	0.2056
MDA (nmol/mL)	CS	2.22 ± 0.390 ^a^	5.42 ± 0.380 ^b^	4.72 ± 0.140 ^c^
	EUE	2.05 ± 0.250 ^a^	5.25 ± 0.250 ^b^	3.66 ± 0.240 ^c^*
*p*-value	/	0.5593	0.5614	0.0029

Symbol * indicates a significant difference at *p* < 0.05 between the *Eucommia ulmoides* group and the control group at the same time. ^a,b,c^ Values with different letters within the same row are significantly different (*p* < 0.05). Values are the mean ± SEM. Results are means of 15 samples obtained from the test chicks of each control and experimental group.

**Table 7:** The immune parameter status of chicks under cold stress.

Index	Group	0 d	7 d	15 d
IgA (μg/mL)	CS	359 ± 4.59 ^a^	409 ± 2.37 ^b^	305 ± 8.19 ^c^
	EUE	353 ± 6.26 ^a^	415 ± 2.86 ^b^*	315 ± 1.74 ^c^
*p*-value	/	0.2477	0.0397	0.1114
IgG (μg/mL)	CS	2419 ± 26.5 ^a^	3069 ± 20.0 ^b^	2109 ± 62.5 ^c^
	EUE	2409 ± 51.7 ^a^	3162 ± 15.3 ^b^*	2389 ± 30.0 ^c^*
*p*-value	/	0.7812	0.0030	<0.0001
IgM (μg/mL)	CS	600 ± 1.70 ^a^	713 ± 5.88 ^b^	846 ± 7.78 ^c^
	EUE	603 ± 4.49 ^a^	721 ± 1.70 ^b^	890 ± 7.40 ^c^*
*p*-value	/	0.3486	0.0907	<0.0001

Symbol * indicates a significant difference at *p* < 0.05 between the *Eucommia ulmoides* group and the control group at the same time. ^a,b,c^ Values with different letters within the same row are significantly different (*p* < 0.05). Values are the mean ± SEM. Results are means of 15 samples obtained from the test chicks of each control and experimental group.

**Table 8:** The organ index of chicks under cold stress.

Group	Organ Index						
	Heart	Liver	Spleen	Bursa of Fabricius	Stomach	Duodenum	Pancreatic
7 d							
CS	7.79 ± 0.370	45.2 ± 0.370	1.57 ± 0.050	3.19 ± 0.050	30.2 ± 3.77	21.3 ± 1.00	3.72 ± 0.660
EUE	7.93 ± 0.220	45.6 ± 0.240	1.55 ± 0.030	3.38 ± 0.080	31.1 ± 0.120	22.2 ± 0.050	3.77 ± 0.030
*p*-value	0.6775	0.2729	0.6460	0.0590	0.7624	0.2763	0.9189
15 d							
CS	7.30 ± 0.770	39.6 ± 0.990	2.39 ± 0.380	3.28 ± 0.050	26.5 ± 5.07	14.5 ± 2.62	3.80 ± 0.500
EUE	7.85 ± 0.360	39.7 ± 0.720	2.87 ± 0.310	3.30 ± 0.010	28.0 ± 1.72	15.0 ± 0.820	4.27 ± 0.650
*p*-value	0.4107	0.9494	0.1912	0.6149	0.6979	0.8016	0.4677

Values are the mean ± SEM. Results are means of 3 samples obtained from the test chicks of each control and experimental group.

## Data Availability

The data that support the findings of this study are available from the corresponding author on reasonable request.
